# Microplastics as benzo-a-pyrene carriers: genotoxicity assessment simulating human gastric digestion

**DOI:** 10.1007/s00204-025-04046-8

**Published:** 2025-04-19

**Authors:** Sebastiano La Maestra, Francesco D’Agostini, Mirko Benvenuti, Stefano Alberti, Mario Passalacqua, Francesca Gronda, Linda Ferrea

**Affiliations:** 1 Department of Health Sciences (DISSAL), University of Genoa, Via A. Pastore 1, 16132 Genoa, Italy; 2 Department of Chemistry and Industrial Chemistry, University of Genoa, Via Dodecaneso 31, 16146 Genoa, Italy; 3 Department of Experimental Medicine (DIMES), Biochemistry Section, University of Genova, 16132 Genoa, Italy

**Keywords:** Microplastics, Pollution, Benzo-a-pyrene, Genotoxicity, Health effects, Gastric digestion

## Abstract

Microplastic particles (MPs) are ubiquitous environmental pollutants that can remain in ecosystems for prolonged periods. Plastic materials undergo various degradation processes driven by chemical, physical, and biological factors that alter their size, shape, composition, and bioavailability. The gastrointestinal tract is the primary pathway through which MPs are absorbed, raising concerns as they can transport harmful pollutants and microorganisms into the body. Despite their widespread presence, the effects of exposure to MPs that vehicle environmental toxins are still not well understood. In this study, we rigorously simulated the photoaging processes of polystyrene MPs of two distinct sizes (1 µm and 5 µm) and confirmed their capacity to adsorb benzo[a]pyrene, a known carcinogen. Moreover, we explored the transport capabilities of these MPs and analyzed their genotoxic effects on liver cells under simulated gastric digestion conditions. Our findings reveal that MPs enriched with BaP release this toxic compound when ingested and exposed to gastric juices, markedly increasing their toxicity compared to the individual components. This research underscores the alarming potential of MPs to exacerbate risks associated with environmental pollutants in human health.

## Introduction

Microplastic particles (MPs) are considered ubiquitous pollutants that can persist in the environment for a long time. When dispersed in environmental matrixes, plastic materials undergo degradation processes triggered by chemical, physical and biological events that determine changes in size, morphology, composition and bio-disponibility. The reduction in the size of plastic material increases its dispersibility in air, water, and soil, increasing damage to the ecosystem and putting a risk to human health (Kibria et al. 2023). Different studies highlight the mechanism undergoing pathological events such as oxidative stress and inflammations related to MPs ingestion and accumulation (Visalli. et al. 2023; Lagana et al. 2024). Current estimates report that a single individual ingests about 74,000 to 121,000 particles/per year and inhales 53,700 particles/per year, being able to absorb even through the skin (Kannan 2021).

Once ingested, MPs can penetrate and accumulate within the epithelial barrier and be distributed throughout the body via the blood or lymphatic system. This process enables MPs to reach critical organs, including the liver, kidneys, and brain (Massardo et al. 2024; Nihart et al. 2025; Zarus et al. 2021).

Moreover, inhaled MPs can be deposited in terminal bronchioles, alveolar ducts, and alveoli, resulting in chronic inflammation, granulomas, or fibrosis (Greim et al. 2001). Additionally, a small fraction of MPs might cross the alveolar wall and enter the capillaries, reaching the bloodstream (Feng et al. 2023), where an immune response that can be local or systemic can be activated (Zhao et al. 2024).

Ingestion is a significant route for exposure to MPs, driven by the prevalence of these particles in food, beverages, and drinking water. When ingested, MPs pass from the oesophagus to the stomach, undergoing a gastric digestion phase lasting 2 to 6 h (Dawson et al. 2018) and then passing to the intestine.

The gastrointestinal tract represents the main route for the absorption of MPs, and the kinetics of accumulation and distribution are influenced mainly by size (Stock et al. 2020). The phenomena that can occur in the intestine can be different. Studies on animals demonstrate that MPs change intestinal permeability and increase inflammation levels, compromising its functionality until chronic diseases are established (Auguste 2021; Hirt and Body-Malapel 2020). The same MPs seem to determine dysbiosis, influence intestinal homeostasis, and tend towards establishing inflammatory pathologies or metabolic disorders (Jin et al. 2019).

After digestion, the MPs absorbed by the intestine can be distributed to different organs and systems. The blood from digestion through the portal vein is conveyed to the liver, the main organ responsible for detoxification. Noteworthy, MPs can act as a vehicle for several pollutants such as polycyclic aromatic hydrocarbons, heavy metals, polybrominated ethers, antibiotics and others (David Grande-Tovar et al. 2022) as well as several microorganisms.

Numerous ecological studies have explored the harmful effects of MPs on various vertebrate and invertebrate species (Jeong 2024). However, the relationship between exposure to MPs and the risk of developing specific disease conditions remains complex and requires further research. Most current studies primarily assess the MPs concentration, size, morphology, and distribution in specific organs (Massardo et al. 2024; Horvatits et al. 2022). The risk attributable to additives such as bisphenol A and phthalates released from plastics during the aging process is controversial (Koch and Calafat 2009; Meeker et al. 2009; Talsness et al. 2009; Wagner and Oehlmann 2009) depending on the type and quantity. While some studies are starting to investigate the potential impacts of xenobiotics absorbed by MPs from environmental sources such as drinking water (La Maestra et al. 2025), the implications for human health are still uncertain (Dzierżyński 2024).

Plastic polymers have the common characteristic of being non-polar and establishing electrochemical interactions with different compounds with which they come into contact, absorbing them. Adsorption processes are favoured by the MPs large surface area and are influenced by environmental conditions, such as the characteristics of the same MPs and the medium in which they are dispersed (Joo 2021).

Research on the adsorption capacities of MPs indicates that polystyrene (PS) has a greater affinity for organic compounds than other plastic polymers. Additionally, the authors note that other factors can play a role in forming π-π interactions (Hüffer and Hofmann 2016). In this context, a study by Gomiero et al. (2018) in *Hediste diversicolor* showed that PVC -MPs have dose-dependent absorption kinetics for benzo-a-pyrene (BaP) and that BaP-enriched MPs exhibit more significant toxicity than MPs alone.

Knowledge about the consequences of exposure to MPs that vehicle environmental pollutants are still underexplored. In this study, we simulated the photoaging processes of PS-MPs at two different sizes (1 and 5 µm) and validated their capacity to absorb apolar organic compounds, such as BaP. Moreover, we tested the transport capabilities of MPs and analyzed their genotoxic effects on liver cells, simulating the conditions of gastric digestion.

The results confirmed that BaP-enriched MPs (MPs_ox-BaP_) release the chemical compound when ingested and exposed to gastric juice, thereby increasing its toxicity compared to the components individually. Understanding the toxicity of these ubiquitous particles and their relationship with xenobiotics is crucial for assessing their potential risks to human health.

## Materials and methods

### Chemical and reagents

All Reagents, such as BaP, fetal bovine serum, antibiotics, cytochalasin B, salts, pepsin, grown medium (EMEM), ethidium bromide and Shiff’s reagent, were purchased by Sigma-Aldrich (St. Louis, Missouri, USA).

### Microplastics oxidation

Virgin MPs of PS with different dimensions (1 and 5 µm), acquired by Cospheric (Santa Barbara, California 93,160 USA), were oxidated as described by La Maestra et al., (2024). Briefly, 1 ml of MPs suspension (10 mg/ml) was dispersed in a glass disc, adding 1 ml of 40% hydrogen peroxide and was exposed to a UVB lamp (318 nm, 57 V, 20 W G13, Philips) for 96 h, periodically agitating. Obtained MPs_ox_ were recovered by centrifugation (12,000 rpm for 10 min), washed in Milli-Q water and dried by SpeedVac (Jouan RC 10.10 Heated Evaporative Centrifuge Concentrator, Milano, Italy), weighted, resuspended in Milli-Q water at known concentrations and resuspended before each use using an ultrasonic device (Bandelin SONOREX™ SUPER, Ultrasonic baths, 35 kHz, Berlin, Germany).

### ***BaP adsorption in MPs***_***ox***_

To allow BaP adsorption in MPs_ox_, 5 mg of the 1 or 5 µm MPs_ox_ were dispersed overnight in 1 mL of a 1 µM BaP aqueous solution into a glass vial under continuous agitation. The 1 µM BaP solution was obtained by solubilizing 2.52 mg of BaP in 1 ml of acetone (10 mM) that subsequently was diluted in Milli-Q water to reach the final concentration. Subsequently, the MPs_ox_ suspension was centrifuged at 12,000 rpm for 15 min to separate the MPs_ox_ from the supernatant, where the unabsorbed BaP was dispersed. The MPs_ox-BaP_ were rinsed and stoked in Milli-Q water for the subsequent tests. The supernatant solution containing BaP was recovered to assess GC–MS.

### Dynamic light scattering

Dynamic Light Scattering (DLS) analysis highlighted UVB photo-aged MPs and MPs_ox-BaP_ surface modifications, as reported by La Maestra (2025). In particular, the ζ-potential, which correlates to the MPs surface charge, as well as the hydrodynamic radius of MPs were measured by DLS analysis using a Zetasizer Instrument, Nano ZS90 Series (Malvern Panalytical, Malvern, UK) equipped with 633 nm He–Ne laser. Briefly, before each measurement, the suspensions of MPs samples (0.1 mg/mL in deionized water) were sonicated (40 kHz) for 5 min, and the analyses were performed at the fixed temperature of 25 °C using a Peltier thermostatic system, with an equilibrating time set to 120 s. Twenty runs, repeated n times (*n* = 3, 6, 9), were performed for each ζ-potential and hydrodynamic radius measurement.

### Cell culture

The human hepatocellular carcinoma cell line (HepG-2) was purchased from the European Collection of Cell Cultures (ECACC). HepG-2 was maintained in a complete culture medium (EMEM, 10% FBS, 10% Glutamine and 1% Penicillin/Streptomycin) at a temperature of 37 °C in a humidified atmosphere with 5% CO_2_ and split every two days or used for the different assays.

### Cells viability test

MTT assay was performed to highlight toxicity effects induced by MPs_ox_ in HepG-2, as reported by (La Maestra et al. 2024). Briefly, 8 replicates were used for each condition. HepG-2 cells were transferred onto 96 multiwell plates (6 × 10^3^ per well), and viability conditions were tested by the 3-(4,5-dimethylthiazol-2-yl)−2,5 dipheny tetrazolium bromide reduction capacity of living cells. Purple formazan products were measured at 570 nm using a multiwell photometer (Multiskan FC, Thermo Fisher Scientific, Waltham, Massachusetts, USA). Viability was expressed as a percentage of absorbance concerning untreated controls. MTT assay was performed at different MP concentrations (25 to 500 µg/ml), sizes and conditions (oxidate or virgin) for 24 and 48 h.

A trypan blue exclusion test was also performed before each assay to assess cell viability. Briefly, the test was performed by mixing10 µL of Trypan blue with 10 µL to discriminate live cells from dead ones. Less than 80% viability was considered an exclusion factor for each test.

### GC–MS

As La Maestra et al. (2025) reported, GC analyses were performed. Briefly, an HP5890 series II gas chromatograph was used coupled to an HP5972 mass spectrometer and equipped with an electron impact ionization source (Hewlett-Packard, Palo Alto, CA, USA).

The adsorptive capacity of MPs_ox_ in an aqueous solution was assessed indirectly by measuring the concentration of residual BaP in the solution after the MPs_ox_ stayed overnight. In particular, after centrifuging the suspension containing MPs_ox_ and BaP at 12,000 rpm for 15 min to separate the MPs_ox_ from the solution, the supernatant was treated with 1 ml of cyclohexane and vortexed for 5 min. The affinity of not adsorbed BaP for cyclohexane allows the total collection of the compound in the organic phase, which is subsequently injected and analyzed in GC–MS.

A standard calibration line was performed by BaP standard samples that were derived from diluting 1 mM BaP stock solution in cyclohexane to final concentrations of 0.1, 1, 5 and 10 µM. Five microliters of each solution were injected into an HP5890 series II gas chromatograph coupled to an HP5972 mass spectrometer equipped with an electron impact ionization (IC) source (Agilent). Three replicates were made for each analysis, and the results were obtained by averaging the values.

GC separation was performed on a DB5MS capillary column (Phenomenex, 0.25 mm × 30 m, 0.25 μm film thickness) set at 1 ml/min, where helium gas flow was used as a carrier. The analyte was volatilized in an injector set at 350 °C, and the column was maintained at an initial temperature of 80 °C. The initial temperature of the oven was set to 80 °C and maintained at an isotherm for 3 min. Subsequently, the oven was heated until 240 °C was reached, at a 30 °C/min gradient (5.3 min total), and then further heated until a temperature of 300 °C was reached, at a 50 °C/min gradient (1.2 min) maintaining at an isotherm for 15 min. After analyzing the BaP standard, mass spectrometry (MS) analysis was performed in SIM (Selected Ion Monitoring) mode, detecting the fragments of interest in the BaP standard. After validation of the calibration line, the BaP recoveries were calculated using the chosen extraction method.

### Gastric juice and MPs digestion

Simulated gastric juice was prepared by dissolving 8.775 g/L NaCl and 1.0 g/L pepsin in Milli-Q water and adjusting to pH 1.3 using 1 N HCl as reported by Kozu et al. (2014).

One hundred microliters of MPs_ox-BaP_ solution (112.5 µg) were incubated with gastric juice (v/v) at 37 °C for 5 h, with constant agitation to simulate gastric digestion. Subsequently, varying volumes of the MPs_ox-BaP_/gastric juice mixture were used to achieve the concentrations reported in the tests below. Notably, the acidic pH of the MPs_ox-BaP_/gastric juice preparation, which was inoculated in small volumes, was effectively neutralized by the buffering capacity of the medium in which the cells were maintained.

### Comet assay

The alkaline comet assay was performed as described by La Maestra et al. 2020 with some modifications. Briefly, the HepG-2 cells were seeded in 96-well plates at the density of 5 × 10^3^ cells/well and exposed to MPs at different conditions (MPs_ox_; MPs_ox-BaP_; predigested-MPs_ox-BaP_) with different sizes (1 and 5 µm) and concentrations (25 and 50 µg/mL)_._ Moreover, BaP to equal concentration adsorbed by 5 and 1 µm MPs_ox_ was used as a positive control.

Subsequently, about 10,000 cells were embedded into 150 μL of 0.7% low melting-point agarose, coated onto slides, previously covered with 1% agar dissolved in PBS, covered with a coverslip and allowed to solidify at 4 °C, followed by a second layer of low melting-point agarose. After a final solidification at 4 °C, the slides were immersed overnight in a cold lysis solution (2.5 M NaCl, 10 mM Tris, 100 mM ethylenediaminetetraacetic acid, pH 10, 1% Triton X-100 and 10% dimethyl sulphoxide). Subsequently, the slides were rinsed in an alkaline solution (1 mM EDTA, 0.3 M NaOH, pH 13) and placed horizontally in an electrophoresis chamber (Bio-Rad, Italy, Milan) in which it was performed in the same fresh alkaline solution, electrophoresis run (30 min at 25 V (0.66 V/cm), adjusted to 300 mA). Obtained slides were washed in a neutralization buffer (0.4 M Tris–HCl, pH 7.5) for 15 min and ethidium bromide-stained (2 µg ml^−1^).

A fluorescence microscope (Olympus BX51) equipped with a digital camera was used to acquire one hundred random nuclei at 200 X. Quantification of DNA damage was performed by CASP software (http://www.casp.sourceforge.net). The results were reported as a percentage of DNA in the tail (%TDNA), and statistical analyses were performed using ANOVA, followed by Bonferroni’s test for the multi-group comparison test. Levels of P < 0.05 were considered significant.

### Cytokinesis-block micronucleus assay

Clastogenic and aneugenic effects were evaluated using the cytokinesis-block micronucleus assay (CBMN) suggested by Fenech (2007). Briefly, 5 × 10^3^ HepG-2 cells were seeded into the chamber slides and, after 24 h, treated under the same conditions reported for the comet assay. After 24 h, 4 µg/mL cytochalasin B was added, and 28 h later, cells were washed, pre-fixed with methanol/acetic acid solution (3:5 ratio) and fixed with methanol/acetic solution (6:1 ratio).

Samples were subjected to acid hydrolysis (HCl 5 N) for 1 h, rinsed in distilled water and DNA stained with Schiff’s reagent (Sigma Chemical Co., St. Louis, MO) for 30 min. After washing them in distilled water, the slides were dipped into running tap water for 5 min to intensify the colouring, and then they were washed and dried for the subsequent microscope observation.

The frequency of micronucleated cells was assessed, scoring about one thousand cells for each sample and using an optical microscope (1000 X). Statistical analyses were performed using Fisher’s exact test to determine a significant difference (*P* < 0.05) between treatments and untreated cells.

### FESEM analysis and confocal microscope

Field emission scanning electron microscopy (FESEM) analysis was conducted to analyze MPs'morphology. Briefly, MPs were vacuum-filtered through a 0.2 μm Whatman polycarbonate filter and allowed to air dry. They were subsequently coated with graphene through sputtering and visualized using a Tescan Clara FE-SEM equipped with a UHR axial beam detector, operating at 5 keV and 300 pA.

HepG-2 cells were seeded onto EZ chamber slides (Merck Group) and treated with MPs_ox_ or MPs_ox-Bap_ for 24 h at a concentration of 25 µg/ml, washed three times with phosphate-buffered saline (PBS), fixed with 2% paraformaldehyde for 5 min at room temperature. Nuclei were stained with 1µM of TO-PRO-3 (ThermoFisher Scientific, Monza, MB, Italy) having strong binding affinity for dsDNA and examined under a Nikon AX R confocal microscope. A PLAN APO λD 60 × OFN25 DIC N2 NA 1.42 oil immersion objective (Nikon Europe B.V. Stroombaan 14, 1181 VX Amstelveen, The Netherlands) was used. Excitation wavelengths/emission bandwidths were microplastic (405/420–500) and TO-PRO (640/649–749). The pinhole size was set to 1 Airy Unit at a wavelength of 488 nm. Images were acquired with 2048 × 2048 pixels and 0.07 µm pixel size. Sequential acquisition was performed to avoid cross-talk between colour channels.

### Statistical analyses

JMP software (version 17. SAS Institute Inc., Cary, NC, 1989–2023) was used for statistical analyses. The multiple individual experiments data were analyzed by one-way analysis of variance (ANOVA) with post hoc testing using the Bonferroni tests. The results were expressed as means ± SD; a *P* value of > 0.05 was considered statistically significant. Fisher's exact test was used to highlight statistically significant differences in the frequency of micronucleated cells between different treatments compared to the control.

## Results

### Dynamic light scattering (DLS)

DLS analysis results obtained from MPs in different conditions (virgin or oxidate) at different sizes (1 or 5 µm), enriched or not with BaP and dispersed in water or a grown medium, are reported in Table 1.

**Table 1:** Hydrodynamic radius size (nm) and ζ-potential (mV) results obtained by DLS analysis in different MPs and environmental conditions

Sample	Condition	BaP	Dispersion medium	Hydrodynamic radius size (nm) ± SD	PdI ± SD	ζ-potential (mV) ± SD
MPs 1 µm	Virgin	*−*	Water	1,058 ± 100.5	0.504 ± 0.43	− 16.0 ± 0.29
	Oxidized	*−*	Water	855.6 ± 149.6	0.406 ± 0.40	− 52.9 ± 1.02
		+	Water	654.5 ± 14.2	0.038 ± 0.01	− 42.3 ± 0.54
		+	Cell medium	1,319 ± 70.3	0.227 ± 0.17	− 24.4 ± 0.94
MPs 5 µm	Virgin	*−*	Water	2,980 ± 723.8	0.283 ± 0.08	− 40.0 ± 1.27
	Oxidized	*−*	Water	1,604 ± 64.1	0.330 ± 0.05	− 40.2 ± 0.70
		+	Water	2,770 ± 147.4	0.393 ± 0.05	− 49.8 ± 3.01
		+	Cell medium	2,556 ± 246.4	0.408 ± 0.08	− 25.3 ± 4.26

Albeit, size distributions by DLS were measured in MPs_v_ and MPs_ox_ in both sizes (1 and 5 µm) and different conditions. The measurements reported in Table 1 were integrated with MPs_ox_ -BaP and dispersed in a growth medium (EMEM). This integration was designed to yield insights into the behaviour of the MPs in the specific conditions that would be utilized for the subsequent in vitro tests. Overall, DLS characterizations highlight surface modifications in PS microparticles triggered by UVB exposure and BaP loading.

In particular, the hydrodynamic radius of 1 µm MPsv overlaps with those reported by the parent company, showing a narrow distribution of the ζ potential around the value of −16.0 mV. Differently, 5 µm MPs_v_ showed a hydrodynamic radius of 2980 nm, which decreased with each subsequent measurement. This effect is probably due to the MPs sedimentation velocity, with ζ potential values of − 40 mV.

The polydispersity index (PdI) related to the measurement of the hydrodynamic radius is less than 0.5 for almost all samples, highlighting a low degree of dimensional dispersion and distribution of the average radius that overlaps with the average size.

The results show how oxidation caused by UVB exposure decreases the average hydrodynamic radius, both for the 1 µm MPs (MPs_v_ 1,058 + 100.5 nm vs MPs_ox_ 855.6 + 149.6 nm) and for the 5 µm MPs (MPs_v_ 2980 + 723.8 nm vs MPs_ox_ 1604 + 64.15 nm), confirming that UVB irradiation affects the surface of the MPs.

Considering the MPs that have been in contact with BaP, it is possible to observe a different behaviour, most likely attributable to the size of the particles. The 1 µm MPs_ox,_ when loaded with BaP, decreases their hydrodynamic radius (654.5 + 14.22 nm) both when compared with the same MPs_v_ (1058 + 100.5 nm) and with the oxidized ones (855.6 + 149.6 nm). The phenomenon mentioned above was not observed in 5 μm MPs, where the hydrodynamic radius of MPs_ox_ (1604 + 64.15 nm) increased after BaP loading (2770 + 147.4 nm).

Furthermore, the oxidation process influenced the surface charge, although it had a greater impact only for 1 μm MPs samples (Table 2). It is worth noting that the dispersion of MPs in different media further modified the particles'chemical-physical characteristics. These changes can be ascribed to the formation of various interactions between different molecules, such as apolar compounds (BaP) or proteins present in the culture medium.

**Table 2:** BaP adsorbed by different sizes of MPs_ox_

Samples	BaP adsorbed by1 mg of MPs_ox_
MPs_ox_ 1 µm	40 ng
MPs_ox_ 5 µm	17 ng

### GC–MS

GC–MS analysis showed that the amount of BaP not adsorbed by MPS_ox_, differs for the two MPS sizes. In particular, 1 μm MPs_ox_ adsorb the 78.8% of BaP, while the 5 μm MPS_ox_ adsorb the 33.3%. This difference in the amount of adsorbed BaP could be due to the higher surface/volume ratio for 1 μm microplastics. Table 2 reports the ng of BaP adsorbed by 1 mg of MPs_ox_.

### Cells viability test

The MTT test results at various concentrations and MPs conditions (25–500 µg/ml) demonstrated no significant effects at any dose or exposure time. Viability values always remained above 85% compared to unexposed cells (Ctrl) and often overlapped with the same control.

### Comet assay

Comet assay performed on HepG-2 highlights genotoxic damage triggered by MPs_ox_ when tested at two different sizes (1 and 5 μm) and concentrations (25 and 50 μg/mL) loaded with BaP and after contact with synthetic gastric juice.

**Fig. 1:**
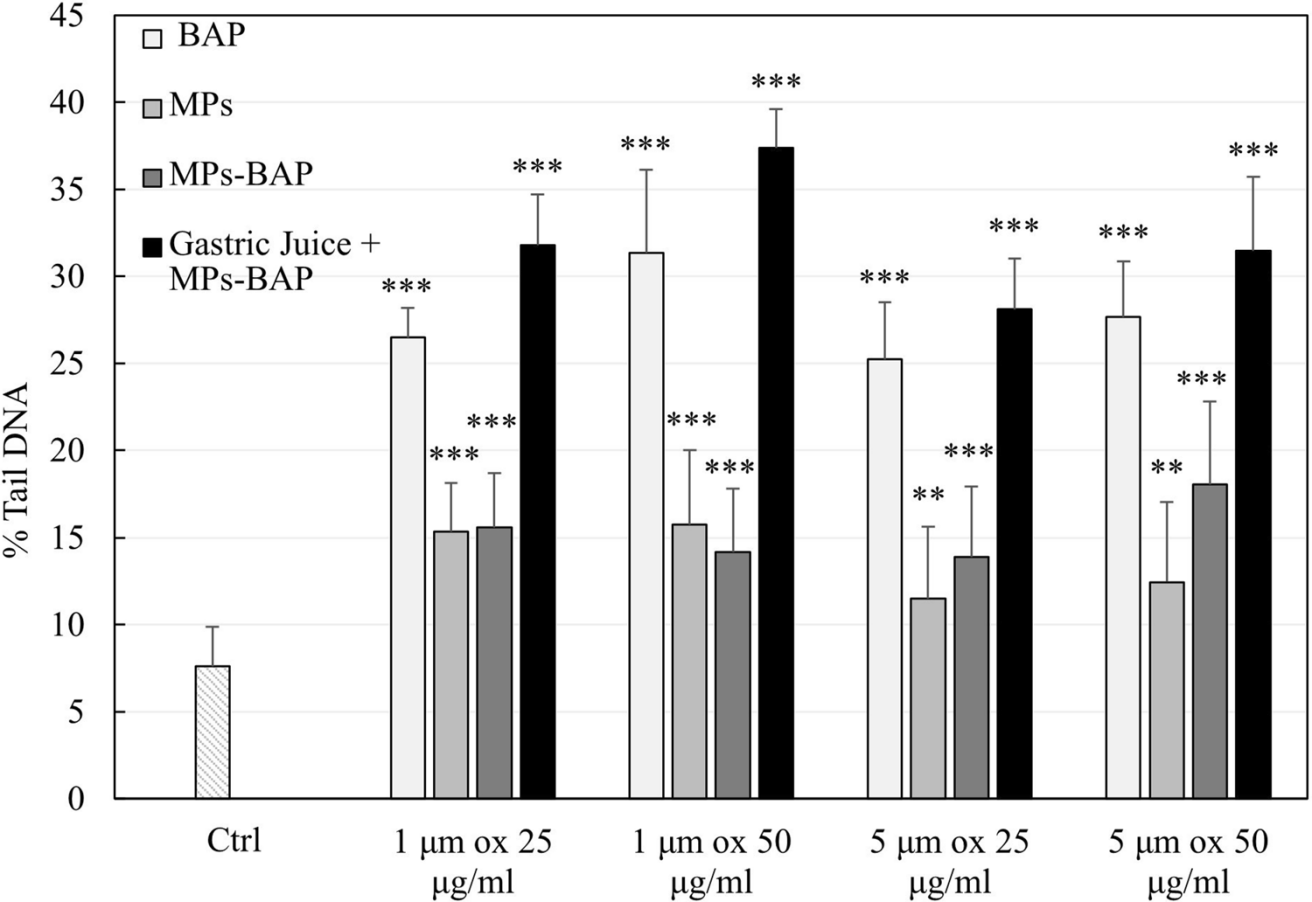
DNA damage (% TDNA) in HepG-2 cells 48 h exposed to 1 and 5 µm MPs_ox_ at two concentrations (25 and 50 µg/mL), with and without BaP loaded and gastric juice treatment or with free BaP. The values are reported as mean + SD. Statistical analysis: **P < 0.01 and ***P < 0.001 compared to the control

The magnitude of genotoxic damage in HepG-2 cells (Fig. 1) expressed as a percentage of tail DNA (% Tail DNA) was compared with an untreated sample (Ctrl). The results show a significant increase in genotoxic damage induced by MPs_ox_ of both sizes and concentrations. Specifically, a two-fold increase in genotoxic damage, compared to the control, was reported for 1 μm MPs_ox_ at both concentrations (*P* < 0.001), while less damage was observed for the 5 μm MPs_ox,_ with a fold increase of about 1.5 for both the concentration (*P* < 0.01). An increase in the % of tail DNA was also observed in cells exposed to BaP (*P* < 0.001), treated with the same concentration adsorbed by the inoculated MPs. The data demonstrate that MPs_ox-BaP_, without gastric juice pretreatment, causes similar damage to MPs_ox_ (*P* < 0.001), while a further increase in damage is observed in cells exposed to MPs_ox-BaP_ previously treated with gastric juice. Specifically, 1 µm MPs_ox-BaP_ treated with gastric juice caused 4.2 times more damage than the control at the lowest concentration (25 μg/mL) and 4.9 times at the highest concentration (50 μg/mL). Differently, the 5 μm MPs_ox-BaP_ treated with gastric juice caused 3.7 times damage compared to the control at 25 μg/mL and 4.1 times at the 50 μg/mL concentration.

### Cytokinesis-block micronucleus assay

**Fig. 2:**
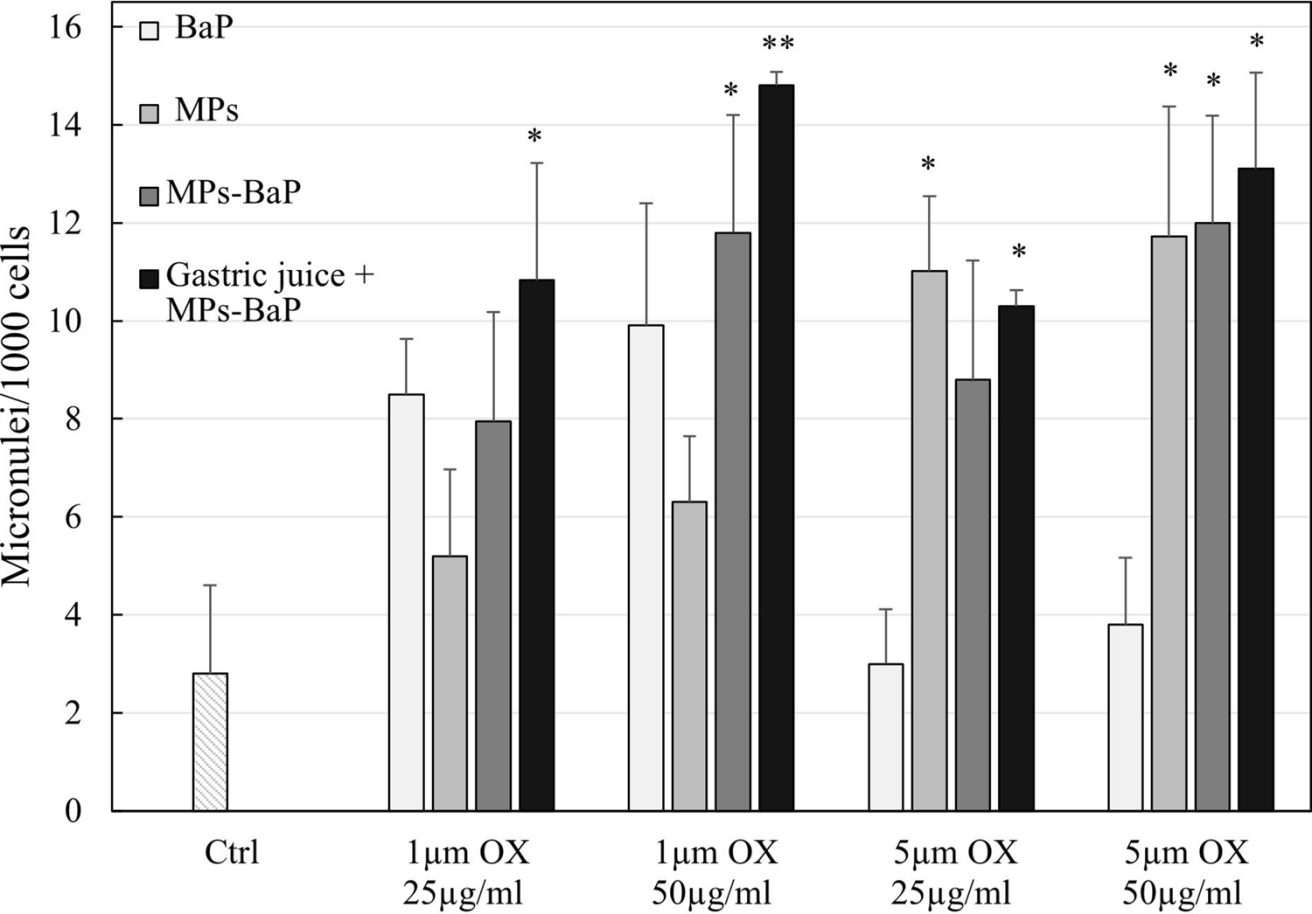
MN frequency in HepG-2 either untreated (Ctrl), exposed to 1 and 5 µm MPs_ox_ at two concentrations (25 and 50 µg/mL), with and without BaP loaded and gastric juice treatment or with free BaP. The values are reported as MN frequency + SD. Statistical analysis: *P < 0.05 and **P < 0.01 compared to the control

Figure 2 reports the frequency of MN observed after different treatments reported above. Overall, exposure to MPs_ox_ caused an increase in MN, although this is not always statistically significant. In particular, no significant increase was observed when HepG-2 cells were exposed to 1 μm MPs_ox_. Differently, 5 μm MPs_ox_ are responsible for the MN frequency increase, reporting fold change value highs to 3.9 (*P* < 0.05).

MPs_ox-BaP_, instead, determined a significant increase in MN frequency at the highest concentration only (50 μg/mL) for both dimensions (a 4.2-fold change for 1μm and a 4.3-fold change for 5 μm) (*P* < 0.05). An additional increase in MN is observed in MPs_ox-BaP_ treated with gastric juice, displaying a dose-dependent response. For the smallest particle size of 1 μm, the fold changes are 3.9 (*P* < 0.05) at a concentration of 25 μg/mL and 5.3 (*P* < 0.01) at 50 μg/mL. For the larger particle size of 5 μm, the fold changes are 3.7 (*P* < 0.05) and 4.7 (*P* < 0.05) at the same concentrations of 25 and 50 μg/mL, respectively.

### FESEM analysis and confocal microscope

**Fig. 3:**
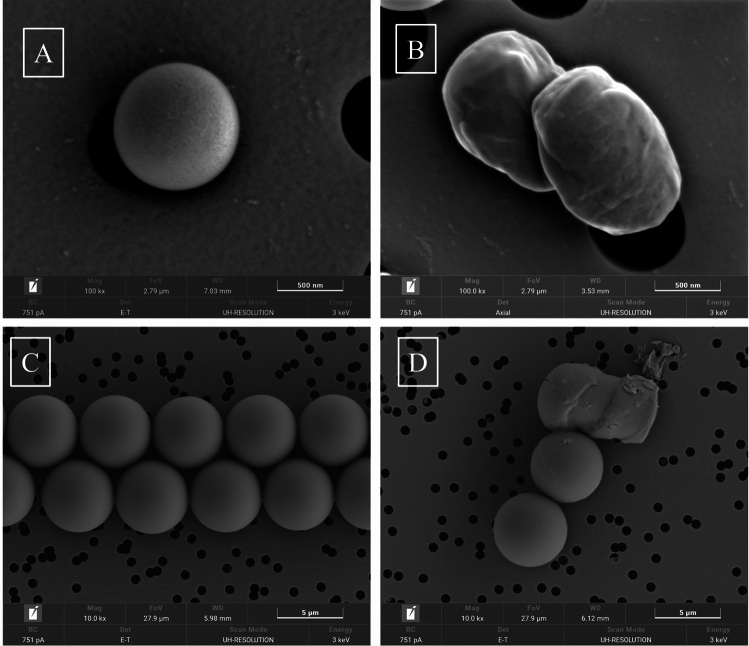
FESEM microphotographs of the MPs 1 μm referred to virgin (**A**) and oxidized (**B**) (100 Kx); MPs_v_ 5 μm (**C**) and MPs_ox_ 5 μm (**D**) (10 Kx), respectively

FESEM analysis performed on MPs samples showed differences between PS in virgin conditions (made by Coospheric) or after photooxidation (Fig. 3). In particular, it is possible to observe in 1 µm MPsox loss of sphericity (Fig. 3B) when compared to MPsv (Fig. 3A) and an increase of surface irregularities attributable to chemical-physical change induced by UVB irradiation. A similar irregularity was appreciated in 5 µm MPsox (Fig. 3D), where, in some cases, it was possible to observe only an increase in the roughness and porosity of the surface.

**Fig. 4:**
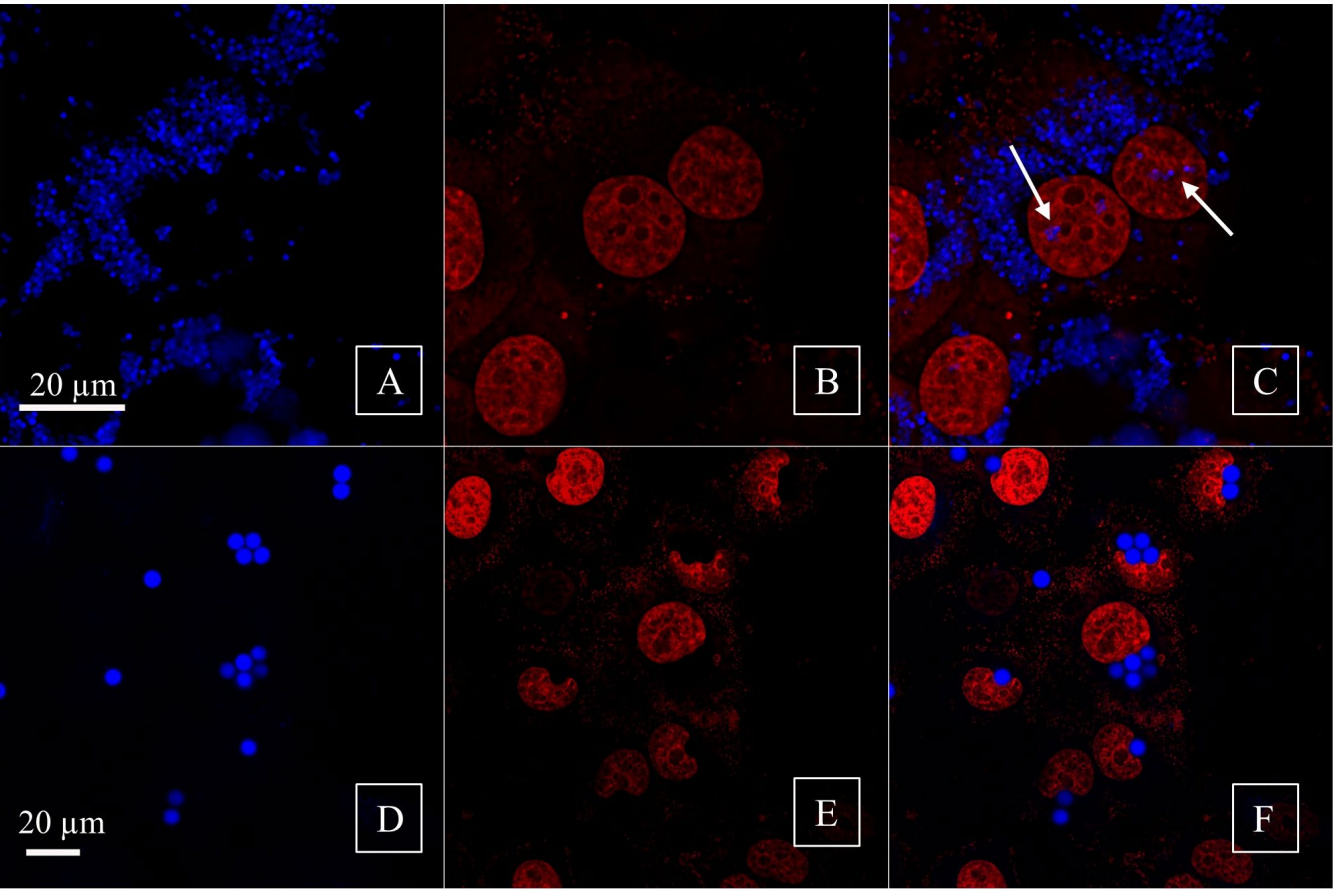
Confocal microscopy was conducted on HepG-2 cells exposed to 1 µm (**A**–**C**) and 5 µm (**D**–**F**) MPs_ox-BaP_, using TO-PRO-3 to label the nuclei. The PS microspheres were excited with a 405 nm wavelength, while the nuclear matrix was excited with a 641 nm wavelength. Panels **C** and **F** display the merged images of the microspheres and the nuclei for both sizes of MPs_ox-BaP_. Different zoom was used to appreciate 5 µm (2) and 1 µm MPs (3.5)

Confocal microscopy analysis confirmed the internalization of MPs_ox-BaP_ in HepG-2 cells. The MPs_ox-BaP_ particles were primarily distributed throughout the cytoplasm for both sizes. Specifically, as illustrated in image 4 C, the 1 μm particles showed a significant presence in the cytoplasm along with small discrete clusters located in the nuclear region, indicated by the arrow in Fig. 4C. In contrast, the 5 μm MPs_ox_ particles displayed an exclusively cytoplasmic localization, which resulted in a deformation of the nuclear compartment's morphology as highlights by the arrow in Fig. 4F.

## Discussions

The present study evaluated, for the first time, the ability of MPs_ox-BaP_, digested by gastric juice, to enhance genotoxic damage in a human hepatocellular carcinoma cell line. The results indicate that MPs_ox_ can absorb organic apolar compounds, such as BaP when dispersed in water and desorb them following contact with gastric juice during digestion.

Processes such as aging, whether through UV irradiation or aging agents like H_2_O_2_ and Fenton reaction, can enhance the adsorption capacity of MPs. (Wang et al. 2019). In particular, we have highlighted that the BaP adsorption in MPs_ox_ depends on particle size. In fact, 1 µm Mps_ox_ adsorb a 2.35-time BaP concentration compared to 5 µm Mps_ox_, probably due to the larger surface-to-volume ratio of the smaller MPs. An additional factor influencing BaP absorption could be due to surface MPs damaging, as FESEM analysis confirms. Photomicrography highlights that 1 µm Mps_ox_ changes shape and surface more than 5 µm Mps_ox_ following the same photoaging treatment.

On the other hand, as reported by results obtained with DLS, a change in hydrodynamic radius size and ζ-potential after UVB irradiation may have influenced the absorptive capacity of the MPs, changing their surface charge. Similarly, this parameter changed when MPs_ox_ was dispersed in different media, such as water or cellular growth mediums or exposed to BaP. The MPs interactions with the cellular system is a complex process that can be affected by the environment in which it occurs. The presence of proteins, pH value, and salt concentration can modify the particle-cell interaction (Wieland 2024).

Polycyclic aromatic hydrocarbons, such as BaP, undergo metabolic processes when they come into contact with an organism. In these processes, enzymes from the cytochrome P450 family oxidize the original compound, forming epoxides and hydroxylated species at various positions. The electrophilic intermediates produced in these reactions can interact with different biological macromolecules, including DNA, resulting in genotoxic effects that may induce carcinogenic processes. BaP is a principal pollutant released by combustion, both natural and anthropic, and when dispersed in the environment, it can be absorbed by MPs_ox._

Using the HepG-2 cell model, which preserves the cytochrome P450 family enzymes, we tested the ability of BaP to trigger genotoxic damage when delivered by MPs_ox_ compared to the effects of BaP inoculated alone. Comet assay, performed in an alkaline environment, detected DNA damage, such as single and double-strand break, and alkaline labile sites, which significantly increased in all tested conditions. Noteworthy, cells treated with MPs_ox-BaP_ reported similar TDNA% values compared to MPs_ox,_ highlighting that BaP, when absorbed into PS, cannot perform toxic effects. Gastric juice pretreatment increases the magnitude of DNA damage, overcoming the effects due to BaP alone exposition. These results suggested that during gastric digestion, following MPs coated with the apolar organic compound ingestion, environmental conditions, such as acid pH and higher temperature, trigger the release of delivered xenobiotics; the same results were discussed by Bakir et al., (2014). Moreover, cells exposed to 1 µm Mps_ox-BaP_ highlight higher DNA damage than 5 µm Mps_ox-BaP._ These results can be due to two different aspects, such as the higher surface/volume ratio, that lead to the increase of BaP adsorption and the ability to reach the cell nuclear area, as confirmed by microphotography obtained by confocal analysis.

Although the comet assay reveals temporary lesions that can ultimately lead to irreversible damage, it is important to recognize that activating repair systems can heal these lesions and restore cellular physiological conditions. Differently, permanent DNA alterations detected by MN assay provide clear evidence of actual damage (Bhagat 2018).

In this context, the MN test revealed a higher damage frequency in cells exposed to 5 μm MPs. This is probably due to the larger size of the particles that, when incorporated, disturb the mitosis processes and alter the nuclear shape, as shown by confocal analysis (Çobanoğlu et al. 2021). Although the 1 μm particles increase the frequency of MN, they do not do significantly, except for the MPs_ox-BaP_ pretreated with gastric juice, where the damage is significant and has a dose-dependent trend. It should be noted that when the cells were exposed to BaP alone, the increase in MN did not reach significant values. This is attributable to the very low concentrations of BaP used to mimic those carried by MPs_ox_.

The set of experimental conditions indeed determined the most significant damage, confirming that the genotoxic damage is undoubtedly corroborated by the physicochemical dualism, such as size, aging state of the MPs, presence of BaP, and environmental conditions of the MPs.

## Conclusion

In conclusion, this study highlights how MPs pollution is an emerging environmental question that must be addressed to find a solution to mitigate the exposure risk. Further research is essential to fully understand the role of microplastics in triggering adverse health effects. However, the worrying ability of these particles to adsorb and release known carcinogens when ingested by humans raises serious concerns for public health.

In this perspective, not only primary prevention strategies aimed at reducing the use and spread of plastic materials in our environment but also implementing effective filtration and purification systems are essential. These systems can significantly reduce exposure and ingestion of microplastics, protecting our communities and future generations from potential harm.

## Data Availability

All experimental data generated and analyzed in this study is included in the published article.

## References

[CR1] Auguste M, Balbi T, Miglioli A, Alberti S, Prandi S, Narizzano R, Salis A, Damonte G, Canesi L (2021) Comparison of different commercial nanopolystyrenes: behavior in exposure media, effects on immune function and early larval development in the model bivalve mytilus galloprovincialis. Nanomaterials (Basel) 11(12):3291. 10.3390/nano11123291 34947640 10.3390/nano11123291 PMC8705110

[CR2] Bakir A, Rowland SJ, Thompson RC (2014) Enhanced desorption of persistent organic pollutants from microplastics under simulated physiological conditions. Environ Pollut 185:16–23. 10.1016/j.envpol.2013.10.007 24212067 10.1016/j.envpol.2013.10.007

[CR3] Bhagat J (2018) Combinations of genotoxic tests for the evaluation of group 1 IARC carcinogens. J Appl Toxicol 38(1):81–99. 10.1002/jat.3496 28695982 10.1002/jat.3496

[CR4] Çobanoğlu H, Belivermiş M, Sıkdokur E, Kılıç Ö, Çayır A (2021) Genotoxic and cytotoxic effects of polyethylene microplastics on human peripheral blood lymphocytes. Chemosphere 272:129805. 10.1016/j.chemosphere.2021.129805 35534956 10.1016/j.chemosphere.2021.129805

[CR5] David Grande-Tovar C, Cesar Carrascal-Hernandez D, Trilleras J, Mora K, Arana VA (2022) Microplastics’ and nanoplastics’ interactions with microorganisms: a bibliometric study. Sustainability-Basel 14:1476. 10.3390/su142214761

[CR6] Dawson AL, Kawaguchi S, King CK, Townsend KA, King R, Huston WM, Bengtson Nash SM (2018) Turning microplastics into nanoplastics through digestive fragmentation by Antarctic krill. Nat Commun 9(1):1001. 10.1038/s41467-018-03465-9 29520086 10.1038/s41467-018-03465-9 PMC5843626

[CR7] Dzierżyński E, Gawlik PJ, Puźniak D, Flieger W, Jóźwik K, Teresiński G, Forma A, Wdowiak P, Baj J, Flieger J (2024) Microplastics in the human body: exposure, detection, and risk of carcinogenesis: a state-of-the-art review. Cancers 16:3703. 10.3390/cancers16213703 39518141 10.3390/cancers16213703 PMC11545399

[CR8] Fenech M (2007) Cytokinesis-block micronucleus cytome assay. Nat Protoc 2(5):1084–1104. 10.1038/nprot.2007.77 17546000 10.1038/nprot.2007.77

[CR9] Feng Y, Tu C, Li R, Wu D, Yang J, Xia Y, Peijnenburg WJGM, Luo Y (2023) A systematic review of the impacts of exposure to micro- and nano-plastics on human tissue accumulation and health. Eco Environ Health 2(4):195–207. 10.1016/j.eehl.2023.08.002 38435355 10.1016/j.eehl.2023.08.002 PMC10902512

[CR10] Gomiero A, Strafella P, Pellini G, Salvalaggio V, Fabi G (2018) Comparative effects of ingested PVC micro particles with and without adsorbed benzo(a)pyrene vs spiked sediments on the cellular and sub cellular processes of the benthic organism Hediste diversicolor. Front Mar Sci 5:99. 10.3389/fmars.2018.00099

[CR11] Greim H, Borm P, Schins R, Donaldson K, Driscoll K, Hartwig A, Kuempel E, Oberdorster G, Speit G (2001) Toxicity of fibres and particles report of the workshop held in Munich, Germany, 26–27 October 2000. Inhal Toxicol 13:737–754. 10.1080/08958370118273 11498804 10.1080/08958370118273

[CR12] Hirt N, Body-Malapel M (2020) Immunotoxicity and intestinal effects of nano- and microplastics: a review of the literature. Part Fibre Toxicol 17(1):57. 10.1186/s12989-020-00387-7 33183327 10.1186/s12989-020-00387-7 PMC7661204

[CR13] Horvatits T, Tamminga M, Liu B, Sebode M, Carambia A, Fischer L, Püschel K, Huber S, Fischer EK (2022) Microplastics detected in cirrhotic liver tissue. EBioMedicine 82:104147. 10.1016/j.ebiom.2022.104147 35835713 10.1016/j.ebiom.2022.104147 PMC9386716

[CR14] Hüffer T, Hofmann T (2016) Sorption of non-polar organic compounds by micro-sized plastic particles in aqueous solution. Environ Pollut 214:194–201. 10.1016/j.envpol.2016.04.018 27086075 10.1016/j.envpol.2016.04.018

[CR15] Jeong E, Lee JY, Redwan M (2024) Animal exposure to microplastics and health effects: a review. Emerg Contam 10(4):100369. 10.1016/j.emcon.2024.100369

[CR16] Jin Y, Lu L, Tu W, Luo T, Fu Z (2019) Impacts of polystyrene microplastic on the gut barrier, microbiota and metabolism of mice. Sci Total Environ 649:308–317. 10.1016/j.scitotenv.2018.08.353 30176444 10.1016/j.scitotenv.2018.08.353

[CR17] Joo SH, Liang Y, Kim M, Byun J, Choi H (2021) Microplastics with adsorbed contaminants: mechanisms and treatment. Environ Chall (Amst) 3:100042. 10.1016/j.envc.2021.100042 37521158 10.1016/j.envc.2021.100042 PMC9767417

[CR18] Kannan K, Vimalkumar K (2021) A review of human exposure to microplastics and insights into microplastics as obesogens. Front Endocrinol (Lausanne) 12:724989. 10.3389/fendo.2021.724989 34484127 10.3389/fendo.2021.724989 PMC8416353

[CR19] Kibria MG, Masuk NI, Safayet R, Nguyen HQ, Mourshed M (2023) Plastic waste: challenges and opportunities to mitigate pollution and effective management. Int J Environ Res 17(1):20. 10.1007/s41742-023-00507-z 36711426 10.1007/s41742-023-00507-z PMC9857911

[CR20] Koch HM, Calafat AM (2009) Human body burdens of chemicals used in plastic manufacture. Philos Trans R Soc Lond B Biol Sci 364(1526):2063–2078. 10.1098/rstb.2008.0208 19528056 10.1098/rstb.2008.0208 PMC2873011

[CR21] Kozu H, Nakata Y, Nakajima M, Neves MA, Uemura K, Sato S, Kobayashi I, Ichikawa S (2014) Development of a human gastric digestion simulator equipped with peristalsis function for the direct observation and analysis of the food digestion process. Food Sci Technol Res 20:225–233. 10.3136/fstr.20.225

[CR22] La Maestra S, Micale RT, Ferretti M, Izzotti A, Gaggero L (2020) Attenuation of oxidative stress and chromosomal aberrations in cultured macrophages and pulmonary cells following self-sustained high temperature synthesis of asbestos. Sci Rep 10(1):8581. 10.1038/s41598-020-65620-x 32444646 10.1038/s41598-020-65620-x PMC7244567

[CR23] La Maestra S, Benvenuti M, Alberti S, Ferrea L, D’Agostini F (2024) UVB-aged microplastics and cellular damage: an in vitro study. Arch Environ Contam Toxicol 87(1):48–57. 10.1007/s00244-024-01073-x 38896243 10.1007/s00244-024-01073-x PMC11283437

[CR24] La Maestra S, Benvenuti M, Gaggero L, Damonte G, Salis A, Alberti S, Ferrea L, D’Agostini F (2025) Environmental microplastics as vectors of non-polar organic pollutants in drinking water. Environmentals 12:81

[CR25] Lagana A, Visalli G, Facciolà A, Saija C, Bertuccio MP, Baluce B, Celesti C, Iannazzo D, Di Pietro A (2024) Sterile inflammation induced by respirable micro and nano polystyrene particles in the pathogenesis of pulmonary diseases. Toxicol Res 13(5):138. 10.1093/toxres/tfae138 10.1093/toxres/tfae138 PMC11368663 39233846

[CR26] Massardo S, Verzola D, Alberti S, Caboni C, Santostefano M, Eugenio Verrina E, Angeletti A, Lugani F, Ghiggeri GM, Bruschi M, Candiano G, Rumeo N, Gentile M, Cravedi P, La Maestra S, Zaza G, Stallone G, Esposito P, Viazzi F, Mancianti N, La Porta E, Artini C (2024) MicroRaman spectroscopy detects the presence of microplastics in human urine and kidney tissue. Environ Int 184:108444. 10.1016/j.envint.2024.108444 38281449 10.1016/j.envint.2024.108444

[CR27] Meeker JD, Sathyanarayana S, Swan SH (2009) Phthalates and other additives in plastics: human exposure and associated health outcomes. Philos Trans R Soc Lond B Biol Sci 364(1526):2097–2113. 10.1098/rstb.2008.0268 19528058 10.1098/rstb.2008.0268 PMC2873014

[CR28] Nihart AJ, Garcia MA, El Hayek E, Liu R, Olewine M, Kingston JD, Castillo EF, Gullapalli RR, Howard T, Bleske B, Scott J, Gonzalez-Estrella J, Gross JM, Spilde M, Adolphi NL, Gallego DF, Jarrell HS, Dvorscak G, Zuluaga-Ruiz ME, West AB, Campen MJ (2025) Bioaccumulation of microplastics in decedent human brains. Nat Med. 10.1038/s41591-024-03453-140164728

[CR29] Stock V, Fahrenson C, Thuenemann A, Dönmez MH, Voss L, Böhmert L, Braeuning A, Lampen A, Sieg H (2020) Impact of artificial digestion on the sizes and shapes of microplastic particles. Food Chem Toxicol 135:111010. 10.1016/j.fct.2019.111010 31794801 10.1016/j.fct.2019.111010

[CR30] Talsness CE, Andrade AJ, Kuriyama SN, Taylor JA, vom Saal FS (2009) Components of plastic: experimental studies in animals and relevance for human health. Philos Trans R Soc Lond B Biol Sci 364(1526):2079–2096. 10.1098/rstb.2008.0281 19528057 10.1098/rstb.2008.0281 PMC2873015

[CR31] Visalli G, Laganà A, Facciolà A, Iaconis A, Curcio J, Pollino S, Celesti C, Scalese S, Libertino S, Iannazzo D, Di Pietro A (2023) Enhancement of biological effects of oxidized nano- and microplastics in human professional phagocytes. Environ Toxicol Pharmacol 99:104086. 10.1016/j.etap.2023.104086 36842547 10.1016/j.etap.2023.104086

[CR32] Wagner M, Oehlmann J (2009) Endocrine disruptors in bottled mineral water: total estrogenic burden and migration from plastic bottles. Environ Sci Pollut Res Int 16(3):278–286. 10.1007/s11356-009-0107-7 19274472 10.1007/s11356-009-0107-7

[CR33] Wang J, Liu X, Li Y, Powell T, Wang X, Wang G et al (2019) Microplastics as contaminants in the soil environment: a mini-review. Sci Total Environ 691:848–857. 10.1016/j.scitotenv.2019.07.209 31326808 10.1016/j.scitotenv.2019.07.209

[CR34] Wieland S, Ramsperger AFRM, Gross W, Lehmann M, Witzmann T, Caspari A, Obst M, Gekle S, Auernhammer GK, Fery A, Laforsch C, Kress H (2024) Nominally identical microplastic models differ greatly in their particle-cell interactions. Nat Commun 15(1):922. 10.1038/s41467-024-45281-4 38297000 10.1038/s41467-024-45281-4 PMC10830523

[CR35] Zarus GM, Muianga C, Hunter CM, Pappas RS (2021) A review of data for quantifying human exposures to micro and nanoplastics and potential health risks. Sci Total Environ 756:144010. 10.1016/j.scitotenv.2020.144010 33310215 10.1016/j.scitotenv.2020.144010 PMC7775266

[CR36] Zhao B, Rehati P, Yang Z, Cai Z, Guo C, Li Y (2024) The potential toxicity of microplastics on human health. Sci Total Environ 912:168946. 10.1016/j.scitotenv.2023.168946 38043812 10.1016/j.scitotenv.2023.168946

